# Ionically Crosslinked Chitosan Membranes Used as Drug Carriers for Cancer Therapy Application

**DOI:** 10.3390/ma11102051

**Published:** 2018-10-20

**Authors:** Alecsandra Ferreira Tomaz, Sandra Maria Sobral de Carvalho, Rossemberg Cardoso Barbosa, Suédina M. L. Silva, Marcos Antônio Sabino Gutierrez, Antônio Gilson B. de Lima, Marcus Vinícius L. Fook

**Affiliations:** 1 Postgraduate Program in Process Engineering, Federal University of Campina Grande, Campina Grande, PB 58429-900, Brazil; alecsandratomaz@hotmail.com; 2 Postgraduate Program in Materials Science and Engineering, Federal University of Campina Grande, Campina Grande, PB 58429-900, Brazil; sandram.carvalho@hotmail.com (S.M.d.S.C); rcbvet@gmail.com (R.C.B.); 3 Department of Materials Engineering, Federal University of Campina Grande, Campina Grande, PB 58429-900, Brazil; suedina.silva@ufcg.edu.br; 4 B5IDA Research Group, Chemistry Department, Simon Bolivar University, Caracas 89000, Venezuela; msabino@usb.ve; 5 Department of Mechanical Engineering, Federal University of Campina Grande, Campina Grande, PB 58429-900, Brazil; antonio.gilson@ufcg.edu.br

**Keywords:** chitosan, pentasodium tripolyphosphate, 1,4-naphthoquinone, ionic crosslinking, controlled release, cancer therapy

## Abstract

The aim of this paper was to prepare, by the freeze-drying method, ionically crosslinked chitosan membranes with different contents of pentasodium tripolyphosphate (TPP) and loaded with 1,4-naphthoquinone (NQ14) drug, in order to evaluate how the physical crosslinking affects NQ14 release from chitosan membranes for cancer therapy application. The membranes were characterized by Fourier transform infrared spectroscopy (FTIR), wide-angle X-ray diffraction (WAXD), scanning electron microscopy (SEM), swelling degree, and through in vitro drug release and cytotoxicity studies. According to the results, the molecular structure, porosity and hydrophilicity of the chitosan membranes were affected by TPP concentration and, consequently, the NQ14 drug release behavior from the membranes was also affected. The release of NQ14 from crosslinked chitosan membranes decreased when the cross-linker TPP quantity increased. Thus, depending on the TPP amount, the crosslinked chitosan membranes would be a potential delivery system to control the release of NQ14 for cancer therapy application. Lastly, the inhibitory potential of chitosan membranes ionically crosslinked with TPP and loaded with NQ14 against the B16F10 melanoma cell line was confirmed through in vitro cytotoxicity studies assessed via MTT assay. The anti-proliferative effect of prepared membranes was directly related to the amount of cross-linker and among all membranes prepared, such that one crosslinked with 0.3% of TPP may become a potential delivery system for releasing NQ14 drug for cancer therapy.

## 1. Introduction

A wide range of materials have been used as drug carriers including, natural and synthetic polymers, lipids, surfactants and dendrimers [1,2,3,4,5,6,7]. However, over the past few decades, many researchers have been committed to producing drug carriers from natural polymers derived from biological systems including protein, DNA, and polysaccharides. This trend is driven not only by the outstanding properties of the natural polymers in terms of biocompatibility, biodegradability, and tissue similarity but also by the growing interest in replacing synthetic petrochemicals by products that are eco-friendly and sustainable [8].

Polysaccharides such as heparin, chondroitin sulfate, and chitosan have been widely investigated as drug carriers [9,10]. Among them, chitosan has gained increasing interest as a material for this application due to its unique characteristics, such as biocompatibility, biodegradability, non-toxicity and ease of multi-functionalization [8,11,12,13,14]. In addition, as a natural biomaterial, it is highly stable, safe and hydrophilic [15,16]. Besides non-toxicity and biocompatibility, chitosan can be degraded in vivo by several enzymes, mainly by lysozyme [17]. Furthermore, the products from degradation are non-toxic oligosaccharides which can be then excreted or incorporated to glycosoaminoglycans and glycoproteins. All these merits have made chitosan a suitable polymer to deliver various drugs and macromolecules [9,18,19,20,21,22]. 

In order to use the chitosan as a drug carrier, inter-chain interactions must be strong enough to form semi-permanent junction points in the molecular network, and the network should promote the access and residence of water molecules inside the polymer network [23]. They can be obtained either by association with small anionic molecules, by hydrogen bonding, by hydrophobic associations, or by polyanions [22]. Among them, ionotropic gelation of chitosan by polyanions, particularly by tripolyphosphate (TPP), a non-toxic polyanion that can interact with chitosan via electrostatic forces to form ionic crosslinked networks, has been widely used [24,25,26,27,28,29,30,31]. Ionically crosslinked chitosan hydrogels can be used as drug delivery systems for controlled release not only in acidic but also in basic media, for rapid release by dissolution and as thermogelling systems. Thus, they have attracted attention for oral sustained drug delivery in the stomach, as well as for intragastric-floating that is used to prolong the retention of the formulations in the stomach [18]. 

Chitosan membranes crosslinked with TPP and designed for pharmaceutical and industrial applications were studied earlier by several research groups [32,33,34,35,36]. However, to the best of our knowledge, up to now TPP-ionically crosslinked chitosan membranes loaded with 1,4-naphthoquinone, a drug that has a variety of pharmacological properties, including antibacterial [37], antifungal [38,39], antiviral [40,41,42], anti-inflammatory [43,44], antiartherosclerotic [45], and anticancer effects [46,47,48,49,50,51,52], has not been reported yet. Hence, in this study, ionically crosslinked chitosan membranes with different contents of pentasodium tripolyphosphate (TPP) and loaded with 1,4-naphthoquinone (NQ14) drug were prepared by the freeze-drying method, in order to evaluate how the physical crosslinking affects NQ14 release from chitosan membranes for cancer therapy applications. The cytotoxic properties of ionically crosslinked chitosan membranes loaded with NQ14 against B16F10 melanoma cell line were also investigated.

## 2. Materials and Methods

### 2.1. Materials

Medium molecular weight chitosan from shrimp shells, *M_v_* = 260 kDa determined by viscometry (PSL Rheotek, São Paulo, Brazil) [53] and degree of deacetylation DD = 90% determined by the infrared spectroscopy (Perkin Elmer, Beaconsfield, UK) method [54], was prepared in the Northeastern Biomaterials Evaluation and Development Laboratory–CERTBIO (Brazil). Pentasodium tripolyphosphate crosslinking agent (TPP, analytical grade), 1,4-naphthoquinone (NQ14) model drug, phosphate buffered saline (PBS) and lysozyme (hen egg-white, HEW) were purchased from Sigma Aldrich (Darmstadt, Germany) and were used as received. Glacial acetic acid from Vetec (Duque de Caxias/Rio de Janeiro, Brazil) and ammonium hydroxide from Neon (São Paulo, Brazil) were analytical grade and used without further purification.

### 2.2. Membrane Preparation

Non-crosslinked and crosslinked chitosan and chitosan/1,4-naphthoquinone membranes were prepared by the freeze-drying and lyophilization technique. To obtain chitosan membranes, 1% (*w*/*v*) chitosan solution was prepared by dissolving chitosan powder in 1% (*v*/*v*) acetic acid (pH 4.16) using a mechanical stirrer operating at 340 rpm and 50 °C for 2 h. Subsequently, this solution (10 mL) was poured into polystyrene Petri dishes (height, 9.87 mm; diameter, 60.35 mm), frozen for 24 h at −84 °C and lyophilized (Liotop L108, Liobras, São Carlos, Brazil) for 72 h. The dry samples were removed from the Petri dishes and neutralized in a 5% (*v*/*v*) ammonium hydroxide (NH_4_OH) for 72 h. Afterwards, samples were washed with distilled water and after another cycle of freezing and lyophilization, microporous chitosan membranes were obtained and coded as CS.

To obtain chitosan/1,4-naphthoquinone membranes (CS/NQ14), 1,4-naphthoquinone powder (118.5 mg) was dispersed into chitosan solution (concentration 750 μmol/L). The prepared solution was sonicated (USC-1400A Ultracleaner ultrasound cleaning bath, Unique, Indaiatuba, Brazil) for 2 h, poured into polystyrene Petri dishes, followed by freezing (24 h at −84 °C) and lyophilization (72 h). Like chitosan membranes, the chitosan/1,4-naphthoquinone membranes dry samples were removed from the Petri dishes and neutralized in a 5% (*v*/*v*) ammonium hydroxide (NH_4_OH) solution for 72 h. Afterwards, they were washed with distilled water and after another cycle of freezing and lyophilization, microporous chitosan membranes were obtained and coded as CS/NQ14.

Both membranes (CS and CS/NQ14) were ionically crosslinked with sodium tripolyphosphate (TPP) by dipping these membranes (1 g) in 0.1%, 0.3% and 0.5% (*w*/*v*) TPP aqueous solution (100 mL) for 5 min. The crosslinking was carried out at room temperature and pH of TPP solution 9.1 (pH of initial TPP solution). After crosslinking, the membranes were repeatedly washed with distilled water, frozen for 24 h at −84 °C and lyophilized for 72 h. Crosslinked chitosan membranes were designed as CS/01TPP, CS/03TPP, CS/05TPP, and crosslinked chitosan/1,4-naphthoquinone membranes as CS/NQ14/01TPP, CS/NQ14/03TPP, CS/NQ14/05TPP. 

### 2.3. Characterization

#### 2.3.1. Fourier Transform Infrared Spectroscopy (FTIR) Analysis

Fourier transform infrared spectra (FTIR spectra) of pentasodium tripolyphosphate (TPP), 1,4–naphthoquinone (NQ14), non-crosslinked and crosslinked chitosan and chitosan/NQ14 membranes were recorded on a Perkin Elmer 400 FTIR Spectrometer (Perkin Elmer, Beaconsfield, UK) equipped with ATR. All spectra were recorded in absorption mode from 4000–600 cm^−1^ with a resolution 4 cm^−1^ and 64 scans.

#### 2.3.2. Wide-Angle X-Ray Diffraction (WAXD)

Wide-angle X-ray diffraction (WAXD) profiles were recorded on a diffractometer Shimadzu model XRD-7000 (Shimadzu, Tokyo/Kyoto, Japan) equipped with a Ni-filtered Cu-Kα radiation. The WAXD profiles were collected in the scattering range 2θ = 5–40°, with resolution of 0.02°, at a scanning speed of 1° min^−1^. The analyses were performed by applying an accelerating voltage of 40 kV and a current intensity of 30 mA. 

#### 2.3.3. Scanning Electron Microscopy (SEM)

Scanning electron microscopy (SEM) was employed to study the surface and cross-sectional morphology of non-crosslinked and crosslinked chitosan and chitosan/NQ14 membranes by using a scanning electron microscope (model TM-1000, Hitachi, Tokyo, Japan). Membranes were mounted on aluminum stubs with double-sided carbon adhesive dots and were sputter coated under vacuum with carbon using Speedivac carbon coating unit (model 12E6/1598, Agar Scientific Limited, Stansted, Essex, England). Images were taken by applying an electron beam accelerating voltage of 15 kV.

#### 2.3.4. Swelling Degree

The swelling degree of non-crosslinked and crosslinked chitosan and chitosan/NQ14 membranes was investigated in phosphate buffered saline (PBS) at various time intervals (over 6 days). The membranes (1 cm × 1 cm) dried at 40 °C for 24 h were weighed initially (*M_d_*) and immersed in phosphate buffered saline (PBS) (pH 7.4) at 37 °C. At predetermined intervals, swollen samples were taken out and blotted off carefully in between tissue papers (without pressing hard) to remove the surface-adhered liquid droplets and then weighed (*M_s_*). Five replicates were performed on each sample. The swelling degree (*SD*) was calculated using Equation (1):

(1) $$SD=\frac{M_{s}-M_{d}}{M_{d}}\times 100$$

where *M_d_* denotes the initial weight of the specimen in its dry state prior to submersion in PBS and *M_S_* denotes the weight of the sample after submersion in PBS (swollen weight) for an arbitrary time interval.

#### 2.3.5. In Vitro Drug Release

The amount of drug released from prepared membranes was measured using an ultraviolet–visible (UV–vis) spectrophotometer (Lambda 35, Perkin Elmer, Beaconsfield, UK). Also, λ_max_ values for the absorbance of NQ14 in PBS (pH = 7.4) were determined by using an UV–vis spectrophotometer. Briefly, freeze-dried membrane sample (154 mg) was incubated in a 25 mL PBS solution (pH = 7.4) and maintained under continuous shaking (speed of 100 rpm) at 37 ± 0.5 °C in an incubator shaker (IKA-KS4000i, IKA, Staufen, Germany). At predetermined time intervals, 3 mL of the release media was taken, and NQ14 release was determined with an UV–vis spectrum analysis at 𝜆_max_ = 341 nm for a period of 360 h. After each measurement, the withdrawn was put back into the system. The experiments were performed in triplicate to minimize the error variation. Average values were used for further data treatment and plotting. The drug concentration was calculated according to a standard curve, and cumulative release was obtained by the following equation:

(2) $$Cumulative\;release\;\left(\%\right)=\frac{\sum_{i=0}^{n}C_{i}V_{0}}{m}\times 100$$

where *V*_0_ is the sampling volume (3 mL), *C_i_* is the concentration (mg l^−1^) of release drug collected at time, and *m* is the mass of the drug incorporated in the polymer (1.185 mg).

#### 2.3.6. In Vitro Cytotoxicity Studies

The cytotoxic effect of NQ14 on B16F10 melanoma cell was measured by MTT assay as described by Price and McMillan [55]. The MTT assay depends on the mitochondrial enzyme reduction of tetrazolium dye to detect and determine cell viability. In brief, the MTT solution (100 μL) was added to 96-well flat bottom microtiter plates at a density of 5 × 10^5^ cells/mL RPMI (Roswell Park Memorial Institute medium) 1640-C per well and incubated at 37 °C under a humidified atmosphere of 5% CO_2_ for 24 h to allow cell adhesion. Afterwards, 200 μL of RPMI 1640-C was then added to each well to dissolve the formazan crystals. Plates were shaken for 5 min on a plate shaker to ensure adequate solubilization. The plate was incubated at 37 °C under a humidified atmosphere of 5% CO_2_ for another 24 h. Then, 100 μL/well of MTT solution (0.5 mg/mL) was added to control and test wells in PBS and incubated at 37 °C for another 3 h period. MTT solution was removed and the purple formazan crystals formed at the bottom of the wells were dissolved using 100 μL isopropyl alcohol/well with shaking for 2 h at room temperature. Absorbance readings on each well were performed at 540 nm (single wavelength) using a Victor 3–Perkin Elmer. Quadruplicate wells were used for each condition and the experiment was repeated thrice. The percentage of cell viability was calculated by Equation (3):

(3) $$\%cell\;viability=\frac{Absorbance\;540\;nm\;of\;treated\;cells}{Absorbance\;540\;nm\;of\;control\;cells}$$

## 3. Results and Discussion

### 3.1. FTIR Analysis

**Figure 1:**
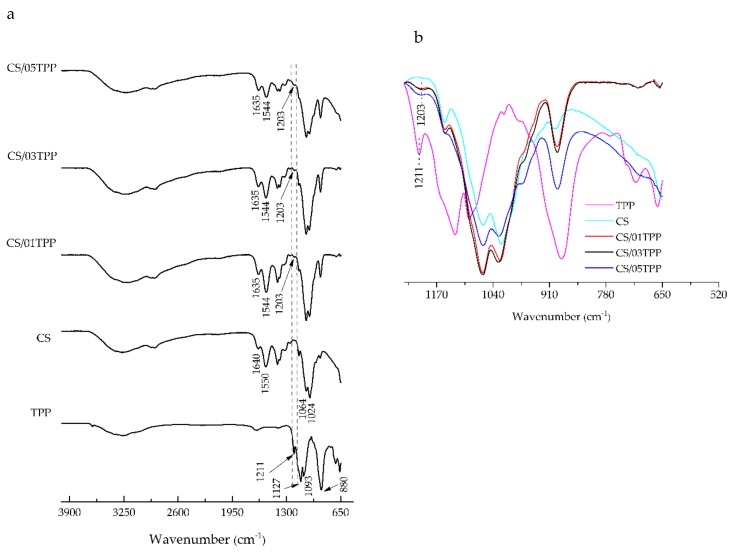
Fourier transform infrared (FTIR) spectra of sodium tripolyphosphate (TPP), non-crosslinked and ionically crosslinked chitosan membranes (**a**) from 4000–500 cm^−1^ and (**b**) from 1245–520 cm^−1^.

**Figure 2:**
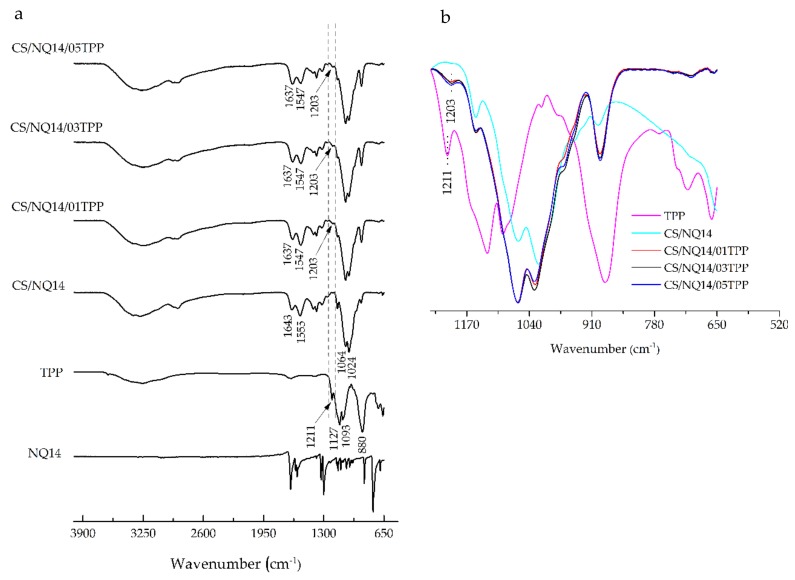
FTIR spectra of NQ14, TPP, non-crosslinked and ionically crosslinked chitosan membranes after NQ14 drug loading (**a**) from 4000–500 cm^−1^ and (**b**) from 1245–520 cm^−1^.

FTIR spectroscopy was used to assess the chitosan chemical groups and to investigate the formation of crosslinked networks from the chitosan with TPP. Furthermore, FTIR spectroscopy was used in order to investigate the evolution of ionic crosslinking associated with the addition of 1,4-naphthoquinone (NQ14) drug. Thus, the FTIR spectra of non-crosslinked and ionically crosslinked chitosan membranes before and after NQ14 drug loading are depicted in Figure 1 and Figure 2, respectively, along with the spectra of the materials separately (TPP and NQ14), which were used as controls in this assay.

In TPP spectra (Figure 1 and Figure 2), the following characteristic bands can be observed: 1211 cm^−1^ (P = O stretching), 1127 cm^−1^ (symmetric and antisymmetric stretching vibrations in PO_2_ group), 1093 cm^−1^ (symmetric and antisymmetric stretching vibrations in PO_3_ group), 800 cm^−1^ (antisymmetric stretching of the P-O-P bridge) [36,56,57]. 

The FTIR spectrum of 1,4-Naphthoquinone (NQ14) drug (Figure 2a) was matched well with the spectra of NQ14 reported in literature [58].

Chitosan membranes spectrum without ionic crosslinking (CS) (Figure 1a), exhibit a broad band in the region of 3500–3200 cm^−1^ that was attributed to O–H and N–H stretching vibrations of functional groups engaged in hydrogen bonds [59,60,61]. Discrete bands were observed around 2900 cm^−1^ and attributed to C–H stretching vibrations [62]. Also, they exhibited characteristic absorption bands at 1640 cm^−1^ (C=O stretching in amide group, amide I vibration), and at 1550 cm^−1^ (N–H bending in amide group, amide II vibration). The bands at the range from 1550 cm^−1^ to 1555 cm^−1^ may be attributed to partially deacetylated amino groups. In other words, there are contributions from both species, protonated amino (–NH) groups and acetyl groups (R–C=O) and confirms that the chitosan is not fully deacetylated. Absorption band at 1322 cm^−1^ (amide III vibration), 1152 cm^−1^ (antisymmetric stretching of the C–O–C bridge due to saccharide structure), 1064 cm^−1^ and 1024 cm^−1^ (skeletal vibrations involving the C–O stretching) are characteristic of chitosan saccharide structure. Likewise, the peak at 893 cm^−1^ due to pyranose ring also confirmed the existing chitosan moiety [59,60,62,63,64,65].

Comparing the spectra of CS and CS/NQ14 membranes (Figure 1 and Figure 2), in the spectrum of CS/NQ14, in addition to the change in the intensity of absorbance, a slight displacement of the characteristic amide I (1640 cm^−1^) and amide II (1550 cm^−1^) bands was noticed. The band at 1640 cm^−1^ shifts to 1643 cm^−1^ and the band at 1550 cm^−1^ shifts to 1555 cm^−1^. These modifications of the spectrum may suggest a possible interaction between chitosan and 1,4-naphthoquinone, as found by Lima, Lia and Ramdayal [32] in a similar study.

After the crosslinking process, in the spectra of all chitosan membranes samples (Figure 1 and Figure 2) appears a new band at 1203 cm^−1^, corresponding to antisymmetric stretching vibrations of PO_2_ groups in TPP ions. This observation indicates the formation of ionic crosslinks between protonated amino groups of chitosan and tripolyphosphate anionic groups, in agreement with the literature [32,34,56,59,66,67]. In addition, the difference in the intensity of the absorption band, assigned to antisymmetric stretching vibrations of the $PO_{2}^{-}$ group in the spectra of chitosan membranes crosslinked with 0.1%, 0.3% and 0.5% (*w*/*v*) TPP solution concentration is observed in the enlarged spectra (Figure 1b and Figure 2b). This result could be attributed to the increment of ionic crosslinking or interpolymer linkage with $-NH_{3}^{+}$ groups of the chitosan by TPP ions. It demonstrated that the ionic reaction of the chitosan-TPP membranes was influenced by the TPP solution concentration.

**Figure 3:**
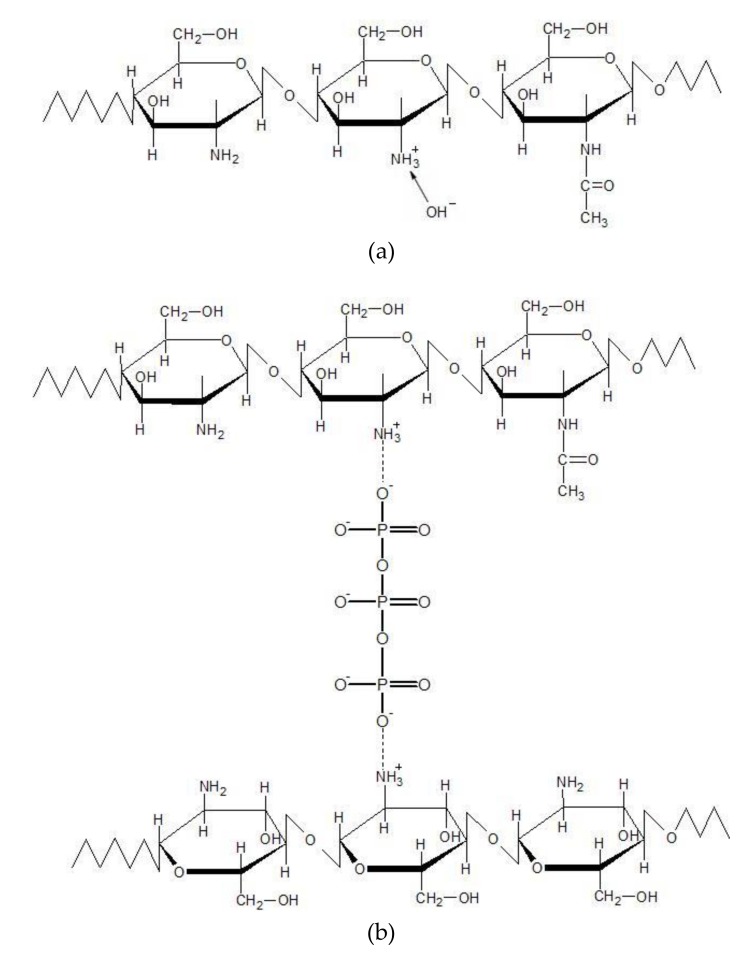
Representation of the $P_{3}O_{10}^{5-}$ and $OH^{-}$ ions reaction with $-NH_{3}^{+}$ groups of chitosan by (**a**) deprotonation and (**b**) ionic crosslinking.

As reported by Mi, Sung, Shyu, Su and Peng [66], TPP dissolved in water dissociates into Na^+^ and tripolyphosphate ions $P_{3}O_{10}^{5-}$, but $P_{3}O_{10}^{5-}$ ions undergo the process of hydrolysis. As a result, when the crosslinking process occurs in TPP solution of basic pH (pH = 9.1) only a slight number of chitosan amino groups is protonated (ionization degree is lower than 5%). In this solution, $P_{3}O_{10}^{5-}$ and $OH^{-}$ ions are present and they can competitively react with $-NH_{3}^{+}$ groups of chitosan by deprotonation and ionic crosslinking, respectively, as presented in Figure 3. 

Lastly, in the spectrum of three components CS/NQ14/TPP membranes (Figure 1), absorption bands at 1203 cm^−1^ and a slight displacement of the characteristic amide I (1640 cm^−1^) and amide II (1550 cm^−1^) observed earlier for two component CS/TPP membranes, can be seen.

### 3.2. WAXD 

**Figure 4:**
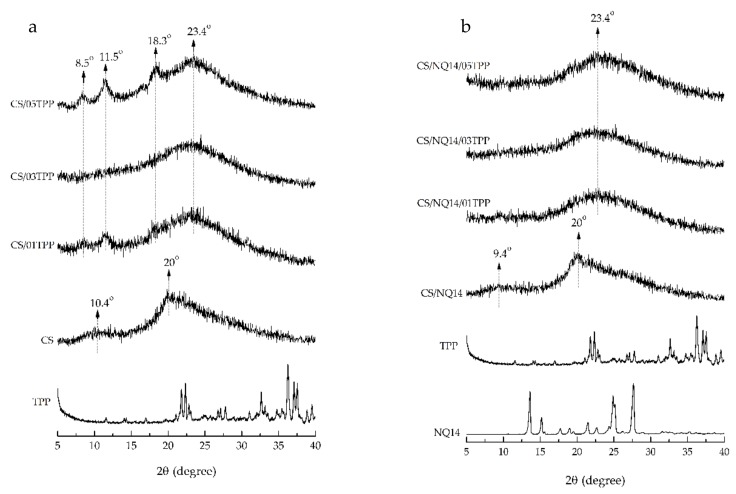
Wide-angle X-ray diffraction (WAXD) profiles of TPP, non-crosslinked and ionically crosslinked chitosan membranes before NQ14 drug loading (**a**); NQ14, TPP, and non-crosslinked and ionically crosslinked chitosan membranes after NQ14 drug loading (**b**).

Ionic crosslinking of chitosan membranes can affect their physical state and, consequently, the drug release behavior. Therefore, WAXD analyses of the non-crosslinked and ionically crosslinked chitosan membranes before and after NQ14 drug loading were performed and the results shown in Figure 4.

Figure 4a shows the WAXD profiles of TPP and non-crosslinked and ionically crosslinked chitosan membranes before NQ14 drug loading. In the X-ray diffractogram of TPP powder, sharp peaks at a diffraction angle of 2θ ≈ 21.8°, 22.4°, 22.8°, 26.7°, 27.1°, 27.8°, 31.0°, 32.6°, 36.2°, 37.0°, 37.5° are present which suggest that the crosslinking agent is present as a crystalline material. The X-ray pattern of non-crosslinked chitosan membrane exhibited a very weak and broad peak at around 2θ ≈ 10.4° and also showed a broad reflection at 20°, indicating the presence of a very small amount of hydrated crystal of chitosan formed by hydrogen bonds among the amino and hydroxyl groups on CS chains [68,69,70,71].

After ionic crosslinking, it is possible to verify that the crosslinked membranes’ diffraction profiles are significantly different, if compared to those of non-crosslinked CS membranes (Figure 4a). The CS/01TPP and CS/05TPP membranes presented higher crystallinity than CS/03TPP ones The CS chains reorganization for CS/01TPP membranes is evidenced by the peaks that appear at 2θ ≈ 8.5°, 11.5° and 18.3°. The high intensity of such new peaks, related to the WAXD profile of raw CS, can be attributed to the large extension of H-bonds among CS–CS, CS–TPP and TPP–TPP chain segments according to Martins, et al. [72]. In addition, the reorganization of CS chains is favored when the used amount of TPP is not enough to neutralize the density of positive charges on CS, that prevail as $-NH_{3}^{+}$ [72]. On the other hand, the large extent of positive charges due to the higher CS/TPP molar ratio on CS/05TPP membranes could induce the appearance of domains with electrostatic repulsion [72]. These repulsion sites could be minimized with structural reorganization process increasing the density of interactions through H-bonds on CS/05TPP membranes. This fact can explain the higher crystallinity of CS/05TPP membranes, as indicated by the higher intensity of the peaks that appear at 2θ ≈ 8.5°, 11.5° and 18.3°, related to CS/01TPP. 

The CS/03TPP membranes, unlike CS/01TPP and CS/05TPP ones, are mostly amorphous, a fact proved by the occurrence of a broad scattering peak at 2θ ≈ 23.4° and by the absence of peaks at 2θ ≈ 8.5°, 11.5° and 18.3°. It is possible that this amount of TPP (0.3 wt %) restrain the movement of the molecular chain of CS due the high crosslinking density preventing the reorganization of CS chains. Since the CS/03TPP membranes are observed in the amorphous state, the release of drugs from these may be better controlled once the higher the amorphization, the higher the swelling ability of the membranes due to the faster relaxation time of the polymeric chains, which may result in an increased drug-release rate [73]. Our results provide evidence that physical state of chitosan membranes was affected by TPP amount used to prepare ionically crosslinked chitosan membranes by immersing them in aqueous TPP solution for 5 min at room temperature and pH 9.1, corroborate with literature data [36,72].

**Figure 5:**
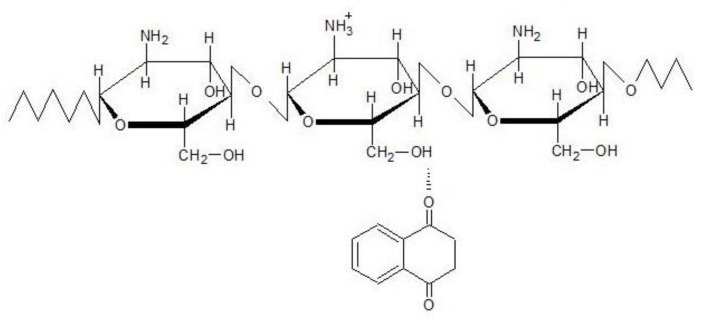
Proposed hydrogen bonding interactions between chitosan and 1,4-naphthoquinone molecules.

Figure 4b shows the WAXD profiles of TPP and NQ14 powders, as well as, of the non-crosslinked and ionically crosslinked chitosan membranes after NQ14 drug loading. Intense diffraction peaks are observed at 2θ ≈ 6.6°, 15.1°, 17.7°, 18.9°, 21.5°, 22.6°, 24.8°, 25.1°, 27.7° in the diffraction pattern of pure NQ14, indicating that NQ14 exists in a crystalline powder state. Although intense peaks were observed in NQ14, only broad diffraction peaks at 2θ ≈ 9.4° and 20° are seen in the combination drug-loaded non-crosslinked CS membranes (CS/NQ14), showing amorphous predominantly nature of this membrane. Likewise, ionically crosslinked chitosan membranes loaded with NQ14 (CS/NQ14/01TPP, CS/NQ14/03TPP and CS/NQ14/03TPP), also show amorphous nature by the presence of a broad scattering peak at 2θ ≈ 23.4°, which corresponds to so-called amorphous halo [68]. These membranes (CS/NQ14/TPP) present two ionic interactions: an intense electrostatic interaction between the positive charged amino group of CS and the negative anion of TPP solution and the electrostatic interactions between hydroxyl groups of CS and oppositely charged NQ14 drug (Figure 5). Thus, the integration of TPP and NQ14 into chitosan disrupted the crystalline structure of chitosan, which is due to the elimination of hydrogen bonding between amino groups and hydroxyl groups in chitosan [74]. Therefore, the drug is observed in an amorphous state in the CS membranes. Similar results were also found by Li, et al. [75] and Papadimitriou, et al. [76] when 5-fluorouracil/leucovorins and pramipexole were encapsulated into CS nanoparticles, in their respective studies.

In summary, the ionically crosslinked CS membranes loaded with NQ14 drug (CS/NQ14/01TPP, CS/NQ14/03TPP and CS/NQ14/05TPP) are more amorphous than the non-crosslinked CS membranes loaded with NQ14 drug (CS/NQ14) (Figure 4b) and for this reason they may present higher drug-release rate. 

### 3.3. Scanning Electron Microscopy (SEM) 

**Figure 6:**
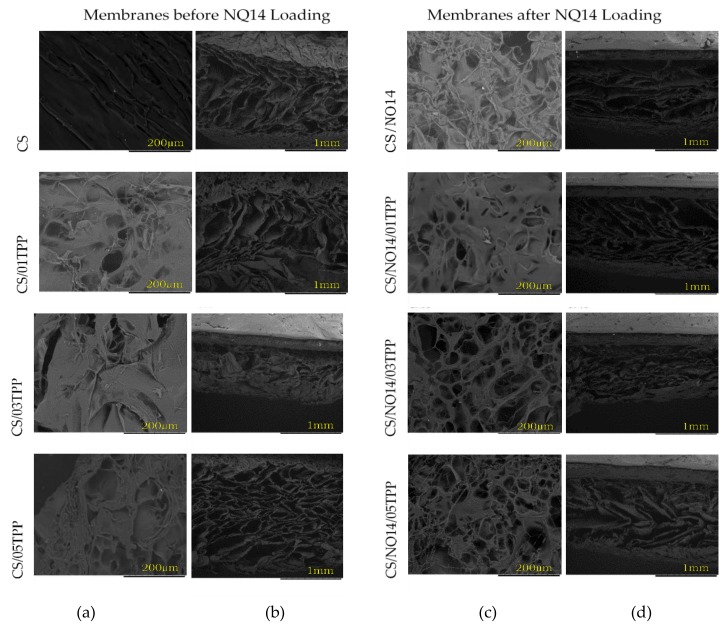
Scanning electron microscope (SEM) photomicrographs of non-crosslinked and ionically crosslinked chitosan membranes before and after NQ14 drug loading, prepared by freezing and lyophilization process: (**a**,**c**) microstructure from the top surface, and (**b**,**d**) from the cross section view.

To investigate the effects of the ionic crosslinking on the structure of the chitosan membranes, which plays a crucial role on controlled release of drugs, the microstructure from both surface and cross section view of non-crosslinked and ionically crosslinked chitosan membranes before and after NQ14 loading are compared in Figure 6.

All membranes possess a three-dimensional interconnected macroporous structure. From the surface view (Figure 6a), the microstructures of non-crosslinked and crosslinked chitosan membranes are quite different. It can be noted that the crosslinked chitosan membranes surfaces were more porous, especially those loaded with NQ14. Open structures with more porosity obtained by crosslinked chitosan loaded with NQ14 could be attributed to higher chitosan chain mobility due to its lower crystallinity, according to previously shown WAXD data (Figure 4). From the cross-sectional view (Figure 1b), open pore microstructure with a high degree of interconnectivity was preserved in all membranes. However, the mean pore size decreased from the non-crosslinked to the crosslinked membranes. No big difference between the crosslinked membranes was observed, except for the one crosslinked with 0.3% of TPP (CS/03TPP and CS/NQ14/03TPP), where elongated pores existed together with condensed walls [77]. The explanation for this observation can be related with the fact that this amount of TPP (0.3 wt %) restrains the movement of the molecular chain of chitosan due the high crosslinking density preventing the reorganization of chitosan chains [78,79,80]. These results show that microstructure such as pore size and its distribution, porosity as well as pore shape is dependent on TPP concentration, and NQ14 presence can influence the controlled release of drugs since it can allow the penetration of biological fluids, facilitating the diffusion of the drugs to the medium.

### 3.4. Swelling Degree

**Figure 7:**
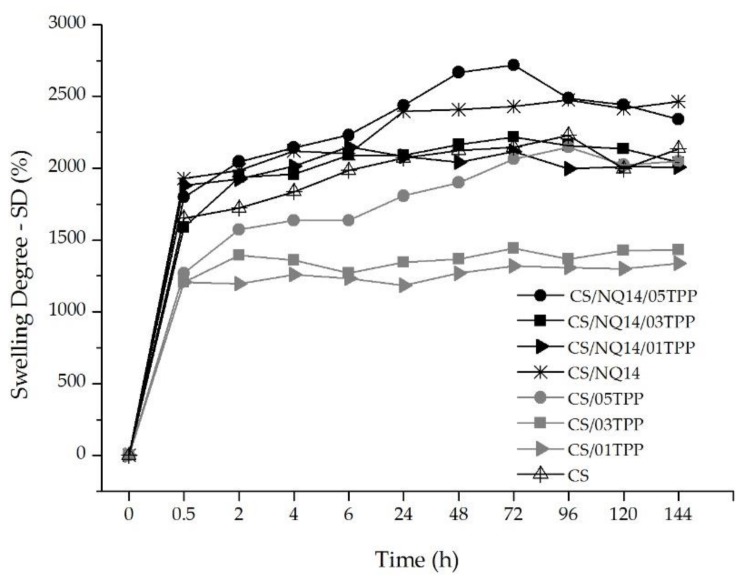
Swelling degree of the non-crosslinked and ionically crosslinked chitosan membranes before and after NQ14 loading with the immersing time.

Swelling characteristics of the crosslinked chitosan membranes are important, since the release of NQ14 from such membranes depends on polymer equilibrium swelling. Thus, swelling capacity of non-crosslinked and ionically crosslinked chitosan membranes before and after NQ14 loading was studied and the obtained results are shown in Figure 7.

According to swelling profiles of CS membranes immersed in buffer solution (pH 7.4) at 37 °C for 144 h, basically 2 stages are observed: an initial rapid mass uptake, usually in approximately 30 min, intrinsically related to volume increase; followed by mass stabilization over a period of 96 h. These results have revealed a strong influence of the ionic crosslinking on the swelling degree, which dropped from approximately 1651% in chitosan membranes before ionically crosslinking (CS) to almost 1207%, 1206%, and 1268%, for ionically crosslinked membranes with 0.1%, 0.3% and 0.5% TPP (CS/01TPP, CS/03TPP and CS/05TPP), respectively, in approximately 30 min. After this time, the CS/01TPP and CS/03TPP membranes swelling degree was essentially the same until 144 h. However, for CS/05TPP membranes the swelling degree increased until 96 h. Although this membrane presents crystallinity degree higher than CS/01TPP and CS/03TPP membranes (as shown in Figure 4), their porosity is higher (Figure 6) resulting in a better water molecules absorption. Ionically crosslinked CS membranes after NQ14 loading (CS/NQ14/01TPP, CS/NQ14/03TPP and CS/NQ14/05TPP) presented the same behavior. Nevertheless, since NQ14 and chitosan are both hydrophilic materials and NQ14-loaded chitosan membranes were more porous (Figure 6), the free space for diffusion increases, allowing these membranes to retain more water molecules inside the network and, consequently, in their higher swelling degree. These results are in good agreement with literature, where it has been reported that, whether the macromolecular chains of a polymer are fixed by ionic crosslinking or chemical crosslinking, the swelling ability of the polymeric networks is reduced [66,81]. The amount of TPP affected the molecular structure, porosity, surface area and the degree of hydrophilicity of the membranes and consequently their swelling behavior.

The swelling is related to a relaxation process among molecules, regardless of whether it is cross-linked or not, and of the possible physical entanglements generated in the membrane-forming process, in this case freeze-drying. For the CS membranes, the greater swelling at pH 7.4 may be associated with the partial neutralization of chitosan, in which the amino groups are protonated, favoring a greater interaction with PBS and the migration of this solution to the preexisting spaces (pores). For the CS/TPP membranes, although the presence of the crosslinking agent reduces this swelling ability, by fixing the macromolecular chains, the neutral pH favors the balance between the charges of the material, promoting maximum interaction among the ionizable groups.

### 3.5. In Vitro Drug Release

**Figure 8:**
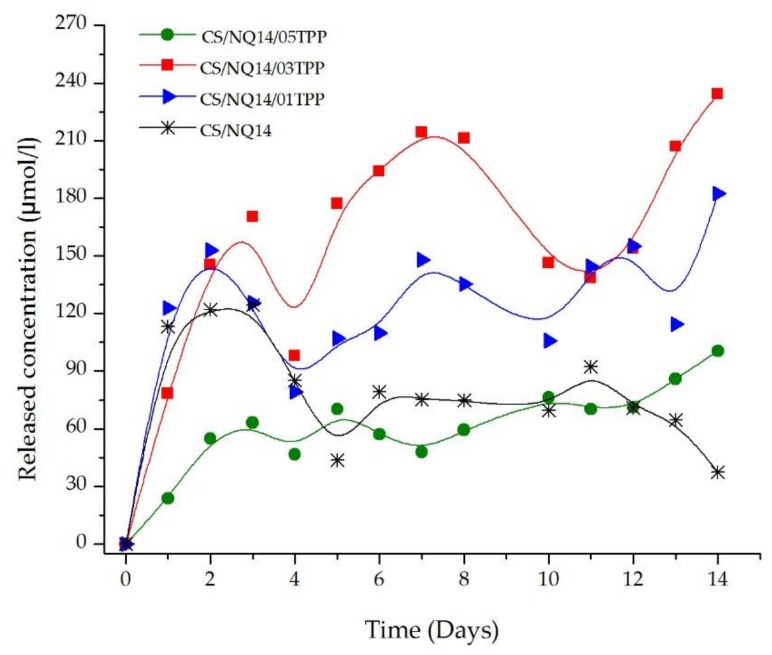
Concentration of NQ14 drug released from non-crosslinked and ionically crosslinked chitosan membranes.

In order to evaluate the influence of the crosslinking agent (TPP) amount on NQ14 drug release from chitosan membranes, in vitro release assays, by UV–vis absorbance, were carried out in phosphate buffer solution (pH 7.4 and 37 ± 0.5 °C) and the results of NQ14 drug release from non-crosslinked and crosslinked chitosan membranes are shown in Figure 8. From this figure, it can be emphasized that drug release rate from chitosan membranes was influenced by the amount of crosslinking agent. The release from membranes having high TPP concentration was slower compared to the release from membranes having low TPP concentration. Typically, the drug diffusion coefficient of a hydrogel decreases as the crosslinking density rises due to increased microstructural tortuosity and decreased space between macromolecular chains [82].

Regarding the initial rapid release, characterized as the “burst effect,” the CS/NQ14/05TPP membrane had the lowest ‘burst’. According to Jameela, et al. [83] and Desai and Park [84], the low amount of surface-bound drug is believed to be responsible for the relatively low burst effect. After this initial burst effect, a slower sustained and controlled release occurred throughout the incubation period, and within 14 days, about 234 µmol/L and 182 µmol/L of the incorporated drug was released from CS/NQ14/03TPP and CS/NQ14/01TPP membranes, respectively. For the membranes having high TPP concentration (CS/NQ14/05TPP) only about 100 µmol/L was released. For the samples prepared without TPP, the amount of NQ14 released from CS matrix was about 37 µmol/L. The higher drug release from CS/NQ14/03TPP compared with CS/NQ14/01TPP can be attributed to a more porous structure (see Figure 6) that is more permeable for the drug. In addition, CS/NQ14 membranes exhibit release profiles similar to CS/NQ1 /05TPP or CS/NQ14/01TPP possibly due to the fact that it was partially neutralized with ammonium hydroxide and that substance associated with NQ14 may have conferred a crosslinking characteristic, a fact observed during the course of the experiment. Thus, crosslinking effectively controls the drug diffusion from chitosan matrix and our results agree with those found by other authors [84,85,86]. 

The results of the in vitro drug release study indicated that the release of NQ14 from chitosan membranes takes place through three steps as depicted in Figure 7. The surface adhered NQ14 is released quickly, 24–122 µmol/L of drug release within 24 h (Figure 7) from all the membranes (step 1). Then, the release rate of the NQ14 from chitosan membranes was sustained which is mainly governed by the diffusion ability of the NQ14 from chitosan membranes into the dissolution media (steps 2 and 3). In addition, it is observed that the concentration decreases and increases randomly due to, probably, the system undergoes into a process of absorption and reabsorption of NQ14. It is possible that the drug at these concentrations can be released and, thereafter, can re-form hydrogen bonds with the surface of the membrane, so it is not available in the solution when used for UV analysis.

It is interesting to note that, although the concentration maximum value of NQ14 reached in this study (234 μmol/L) does not represent a significant portion compared to the incorporated initial quantitative (750 μmol/L), it was a value close to reference as reported by Kayashima, Mori, Yoshida, Mizushina and Matsubara [47]. According to these authors, the concentration of 100 μmol/L of NQ14 was effective in anti-angiogenic action in rats experiments and, at the concentration of 300 μmol/L showed significant inhibition in the growth of HCT116 proliferative cells (human colon cancer cells).

### 3.6. Cell Viability of NQ14-Loaded Chitosan (CS) Membranes against Murine Melanoma Cell Line

**Figure 9:**
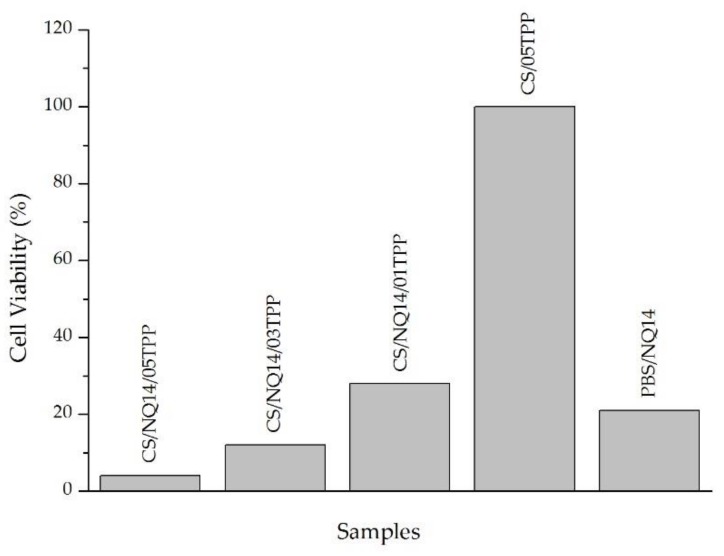
Effect of 1,4-naphthoquinone (NQ14) on the proliferative growth of B16F10 melanoma cell line determined by MTT assay. Membranes loaded with NQ14 (CS/NQ14/05TPP, CS/NQ14/03TPP and CS/NQ14/01TPP), control (membrane without drug-CS/05TPP) and phosphate buffered saline (PBS)/NQ14 control.

MTT assay was employed to investigate the anti-proliferative effects of 1,4-naphthoquinone (NQ14) incorporated into ionically crosslinked CS membranes. B16F10 melanoma cell line was used for this test. Our results (Figure 9) demonstrated that NQ14-loaded crosslinked CS membranes (CS/NQ14/05TPP, CS/NQ14/03TPP and CS/NQ14/01TPP) vigorously hampered the cell viability compared to NQ14-unloaded crosslinked CS membrane (CS/05TPP) used as control. This indicates that NQ14 is a potent inhibitor of melanoma cell growth and shows the efficacy of CS/TPP membranes in carrying the drug inside the cells. Although the three membranes (CS/NQ14/05TPP, CS/NQ14/03TPP and CS/NQ14/01TPP) have showed high cytotoxic activity, the viability of cells treated with NQ14 loaded in CS/05TPP and CS/03TPP membranes fell to less than 15%, indicating the greater efficacy of these membranes in carrying the drug inside the cells. These results suggest that these samples can be considered promising for cancer therapy. In addition, since anti-proliferative agents, such as clinically used anti-cancer agents, suppress angiogenesis by inhibiting endothelial cell proliferation [87], NQ14 would be a potent anti-angiogenic agent. Our results agree with those found by other authors [47,48,88,89]. 

## 4. Conclusions

Ionically crosslinked chitosan membranes with different contents of pentasodium tripolyphosphate (TPP) and loaded with 1,4-naphthoquinone (NQ14) drug were prepared, by the freeze-drying method, and the effect of the physical crosslinking on NQ14 release from chitosan membranes was evaluated. According to the results, the molecular structure, porosity and swelling degree of the chitosan membranes were influenced by TPP concentration and, consequently, the NQ14 drug release behavior from these membranes was also affected. The membranes prepared with the smallest quantities of TPP (0.1% and 0.3%) presented the NQ14 highest rate release and the highest concentration of NQ14 released, especially the ones prepared with 0.3% of TPP and, based on the concentration maximum value of NQ14 released, these membranes can be said to inhibit angiogenesis. To prove the inhibitory potential of these membranes against the B16F10 melanoma cell line, an MTT assay was performed. This assay displayed that anti-proliferative effect of prepared membranes was directly related to the amount of cross-linker. This study suggests that among all membranes prepared, that crosslinked with 0.3% of TPP may become a potential delivery system for releasing of NQ14 drug for cancer therapy.
